# The role of simulation in intertemporal choices

**DOI:** 10.3389/fnins.2015.00094

**Published:** 2015-03-31

**Authors:** Garret O'Connell, Anastasia Christakou, Bhismadev Chakrabarti

**Affiliations:** Centre for Integrative Neuroscience and Neurodynamics, School of Psychology and Clinical Language Sciences, University of ReadingReading, UK

**Keywords:** temporal discounting, reward, empathy, simulation, intertemporal choice, autism

## Abstract

One route to understanding the thoughts and feelings of others is by mentally putting one's self in their shoes and seeing the world from their perspective, i.e., by simulation. Simulation is potentially used not only for inferring how others feel, but also for predicting how we ourselves will feel in the future. For instance, one might judge the worth of a future reward by simulating how much it will eventually be enjoyed. In intertemporal choices between smaller immediate and larger delayed rewards, it is observed that as the length of delay increases, delayed rewards lose subjective value; a phenomenon known as temporal discounting. In this article, we develop a theoretical framework for the proposition that simulation mechanisms involved in empathizing with others also underlie intertemporal choices. This framework yields a testable psychological account of temporal discounting based on simulation. Such an account, if experimentally validated, could have important implications for how simulation mechanisms are investigated, and makes predictions about special populations characterized by putative deficits in simulating others.

## Introduction

Just as our own feelings determine our preferences when making choices for ourselves (Bechara and Damasio, 2005), our understanding of the feelings of others determines how we predict what they would prefer (Nicolle et al., 2012). Similarly, we often make choices on behalf of our future selves. For instance, when choosing between rewards now or for later (known as intertemporal choices), we might assess the worth of the later reward by predicting how much we will enjoy it in the future (Loewenstein, 1996). A possible route to predicting the feelings of others or the feelings of our future selves is by imagining how we would feel in their places, e.g., how a reward would make *me* feel *then*. This mechanism, known as simulation, has been proposed to play a role in predicting the feelings of others and one's own self in the future (Buckner and Carroll, 2007; Mitchell et al., 2011), but how these forms of simulation are related remains to be directly investigated. In this article, we review the experimental evidence for the role of simulation of future selves in making intertemporal choices, and present a heuristic to illustrate its similarity with simulation of others.

### Measures and models of intertemporal choice

Preferences in intertemporal choices can be indexed by the temporal discounting task. In this paradigm, a series of choices between smaller/sooner and larger/later monetary amounts are presented. The commonly observed response pattern is that the longer the delay of the larger reward, the more the sooner but lesser reward is preferred. The rate at which rewards are subjectively devalued as a function of delay slows down as the delays become longer, resulting in a steep-to-flat “discounting curve” (Ainslie, 1975). This discounting function has been associated with intelligence (Mischel and Metzner, 1962; Kirby et al., 2005; Shamosh et al., 2008) and consequential life outcomes such as health, wealth and social-functioning (Mischel et al., 1989; Moffitt et al., 2011).

Several psychological accounts of intertemporal choice have been proposed, such as ego depletion (Baumeister and Heatherton, 1996), hot vs. cool systems (Metcalfe and Mischel, 1999), construal-level models (Fujita, 2008), and empathy-gap models (Loewenstein, 1996). The model proposed in the current paper stems from the empathy-gap model, which suggests that difficulties in empathizing with the feelings of reward belonging to one's future self cause rewards to be devalued with delay. Simulation has been proposed to be an integral component of empathy, in that it allows us to predict another person's mental state by putting ourselves in their shoes (Gordon, 1986; Shanton and Goldman, 2010).

If simulation is used to infer the feelings of future selves, then delayed rewards should be preferred less when simulation is low, as is the case for socially distant strangers. To test this prediction, 63 participants made intertemporal choices from the perspective of socially close and distant others, as well as from their own perspective (O'Connell et al., 2013). We observed that participants preferred delayed rewards less for socially distant others, than for socially close others. This reduction in preference for future rewards for strangers was arguably due to the lower simulation elicited by strangers compared to one's self/friends. Additionally, individuals who scored higher in trait empathy were found to discount less steeply for strangers, suggesting that delayed reward choices are promoted by dispositional markers related to simulation. In the following sections, we discuss simulation and its subcomponents in greater detail, and illustrate a potential role for them in intertemporal decisions.

It is worth noting that this framework operates on a different level of explanation than value-based computational approaches to intertemporal decision-making. For example, a previous framework developed by Pezzulo and Rigoli (2011) also postulates the role of future prospections in intertemporal choices, but concentrates exclusively on the inputs and outputs of value computations. Instead, this article focuses on the putative psychological mechanisms that underlie intertemporal choices.

### Simulating future selves

Evidence from functional magnetic resonance imaging (fMRI) studies highlights simulation as a modulator of **intertemporal preferences**. In a study by Mitchell et al. (2011), participants were asked to imagine rewarding events now or in the future. A reduction in activity in the ventromedial prefrontal cortex (vmPFC, reliably activated when people put themselves in the shoes of another person; Ochsner et al., 2004) was observed when thinking about future rewarding events compared to those in the present. Smaller reductions in this vmPFC response were associated with greater preference for delayed rewards on a temporal discounting task a week later. The authors interpreted this finding to suggest that the extent to which future rewards are simulated (imagining what it would be like to receive them, as if available now) is reduced by delay, and these reductions guide preferences for delayed rewards.

KEY CONCEPT 1 **Intertemporal preferences**

An individual's preference between smaller/immediate rewards or larger/delayed rewards, as indexed by the temporal discounting task.

In a similar study by Peters and Büchel (2010), intertemporal choices were presented concurrently with a description of an event planned by the participant at a similar point in time as the delayed reward. Cueing participants to think about future states arguably induced simulation of future selves, and this was found to increase preferences for delayed rewards.

Lesions studies further suggest the vmPFC's involvement in simulating future rewards. In a study by Sellitto et al. (2010), patients with lesions to the vmPFC (orbital aspect) were reported to prefer immediate rewards more compared to controls. This finding is argued to support the vmPFC's role in imagining/simulating future rewards, and thus, damage to this region reduces preferences for rewards that are more difficult to imagine, i.e., those further away in the future (Peters, 2011; Sellitto et al., 2011). Notably, it has been reported that lesions to the vmPFC also impair the ability to infer another person's feelings (but not their beliefs) (Shamay-Tsoory et al., 2006; Shamay-Tsoory and Aharon-Peretz, 2007). Together, these findings indicate the joint role of the vmPFC in simulating the affective perspectives of both future selves and other people.

The current proposal that future selves are simulated in intertemporal decisions dovetails with work on *episodic future thinking*, i.e., the imagining of future personal events. A theoretical account from Schacter et al. (2007) proposes that episodic future thinking involves simulation, in how future events are imagined by reconstructing past experiences. This account has been supported by findings indicating an overlap in brain regions involved in remembering the past events and imagining future events. For instance, amnesic people with hippocampal damage are disrupted in their ability to episodically imagine future events (Hassabis et al., 2007; Race et al., 2013). Intriguingly, this group show normal levels of temporal discounting (Kwan et al., 2012, 2013; Palombo et al., 2014), suggesting that simulation mechanisms involved in intertemporal choices are less dependent on contributions from episodic memory than those involved in episodic future thinking.

In commonly used measures of episodic future thinking (e.g., past-future task; Addis et al., 2008), participants are asked to mentally construct hypothetical events. Due to the highly specified level of detail in these events, simulating them potentially draws on the hippocampal store of episodic memories to provide ready schemas for constructing these details (Martin et al., 2011; Addis and Schacter, 2012). In contrast, simulation of future selves in intertemporal choices might not require such detail to generate the required signal (i.e., subjective value of choice). In this case, only the feelings associated with receiving rewards need to be simulated, without the need to fully construct the episodic details of the future event in which rewards will be received. This route would preclude the need for major contributions from episodic memory systems. Instead, these simulations could rely upon more generalized affective representations of rewards, possibly signaled in the vmPFC. This view is supported by evidence for the dissociation between systems involved in the cued anticipation of rewards and episodic memory (Packard and Knowlton, 2002). Consistent with its proposed similarity to intertemporal choices, the ability to infer the mental states of others is also preserved following loss of hippocampal functioning (Rosenbaum et al., 2007).

### Simulating others

**Figure 1:**
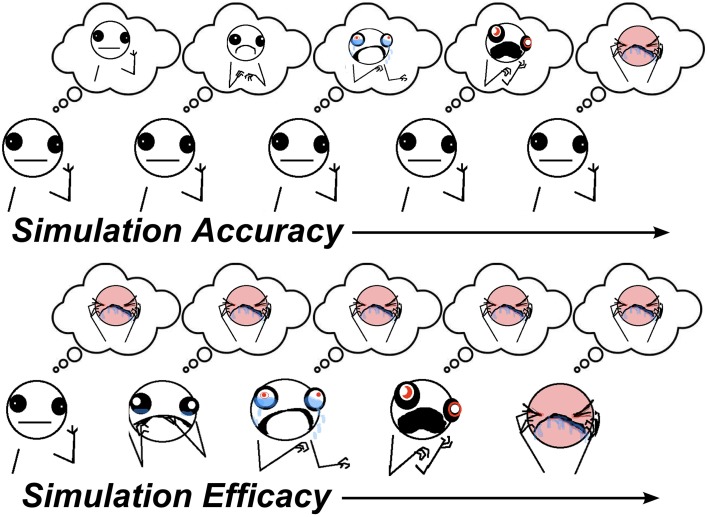
**Visualisation of simulation components. Top panel:** as SA increases, the other's inferred perspective moves from an inaccurate neutral state (caused by egocentric bias to one's own neutral state) toward their actual pain state. **Bottom panel**: as SE increases, the other's predicted perspective becomes simulated in one's self, leading to increased state-sharing between self and other (illustrations by Allie Brosh (hyperboleandahalf.blogspot.com/), published with permission of partial reprint from artist).

Simulation is a theoretical mechanism for how the thoughts and feelings of others are inferred, as in Theory-of-Mind (ToM) tasks (Shanton and Goldman, 2010). It states that to understand others, we put ourselves in their shoes to see the world from their perspective. There are two component processes involved in simulation. In one component, one's own perspective/belief state needs to be adjusted to match the perspective of the other person. A key prediction of simulation accounts is that if one's own perspective is not sufficiently adjusted, this will bias predictions about the perspectives of others toward one's own, an error known as **egocentric bias**. This component of simulation involving suppression of the egocentric bias will hereafter be referred to as **simulation accuracy** (SA) which might involve multiple processes, such as executive function, inhibitory control, and working-memory. The other component of simulation describes the extent to which the thoughts and feelings of others are actively embodied in one's self, and is hereafter referred to as **simulation efficacy** (SE). In this sense, SE is conceptually similar to emotional contagion (Hatfield et al., 1994). SA and SE can be engaged to different extents in the same social cognitive task, depending on task demands. A graphical depiction of these components during simulation is provided in Figure 1. A challenge for lab-based tests is to identify proxy processes for each of these components of simulation.

KEY CONCEPT 2 **Egocentric bias**

The extent to which self perspective interferes with the inference of another's perspective.

KEY CONCEPT 3 **Simulation accuracy**

The extent to which egocentric bias is suppressed in order to make more accurate inferences of others' perspectives.

KEY CONCEPT 4 **Simulation efficacy**

The extent to which the other's perspective is simulated as if happening to the self.

The SA component of simulation has traditionally been measured using the “false-belief” ToM task, which requires participants to suppress their own knowledge in order to infer the minds of naïve others (Wimmer and Perner, 1983; Baron-Cohen et al., 1985). Another measure of SA is the Director task, in which the ability to suppress egocentric bias is operationalised as how much participants inhibit the tendency to respond to task instructions from their own visual perspective, and instead respond according to the visual perspective of another person (Keysar et al., 2003; Epley et al., 2004).

SE has been measured using a range of techniques. In one behavioral approach, it has been shown that observation of emotion states in others leads to a corresponding bodily representation (e.g., facial expression) being activated in the observer (Niedenthal, 2007). The extent of this bodily mapping from the expresser to the perceiver is a proposed metric of SE, in how it also potentially signifies the simulation of others' affective states.

This “state sharing” has also been measured using fMRI techniques. Singer et al. (2004) developed a “self-other pain” paradigm, in which participants' neural responses are measured while they received mild electric shocks, or observe/infer the pain experienced by another person being administered shocks. Singer et al. (2004) reported extensive overlap in neural responses to pain in one's self, and when observing pain in others. The magnitude of neural responses to the pain of others within this overlap has been positively correlated with participants' ratings of the intensity of the others' pain (Cheng et al., 2007; Saarela et al., 2007), directly linking this activity to inferences about the other's perspective. A similar self-other overlap has been noted when making reward chooses on behalf of others in the vmPFC (Nicolle et al., 2012; Suzuki et al., 2012; Janowski et al., 2013; Jung et al., 2013; Morelli et al., 2015), suggesting that in order to predict the preferences of others, we simulate them in ourselves. In sum, neural activity in the self-other overlap when observing/inferring the perspectives of others appears to denote that aspects of the others' experience are being simulated; the SE component of simulation.

Many investigations of SE have examined the effects of changes in social perception. Most notably, studies have contrasted levels of SE elicited by friends compared to strangers, by liked compared to disliked others, or by familiar vs. unfamiliar others. All of these manipulations effectively alter the **social distance** of the simulation target. Social distance is a construct for measuring how close another person generally feels across a range of dimensions, e.g., how familiar, self-similar or socially liked a person is perceived to be (Liviatan et al., 2008; Osiński, 2009). These dimensions can be considered as proxies for the subjective value of the social target, which modulates the motivation for social affiliation. Interestingly, the location of others on these dimensions of social distance is shown to modulate both SA and SE components of simulation, but in opposite directions.

KEY CONCEPT 5 **Social distance**

Perception of another person's position on a general dimension of social closeness, encompassing kinship, familiarity, self-similarity, and likeability.

### Effects of social distance on simulation efficacy

Using the self-other pain paradigm, neural markers of simulation are shown to be more strongly elicited by people perceived as more friendly (Singer et al., 2006), and for loved ones compared to strangers (Cheng et al., 2010) (see Table 1 for further examples). Similarly, neural responses to rewards for others in the self-other reward overlap are found to be greater for socially close vs. distant others (Table 1). Beyond pain and reward, studies also demonstrate increased neural activity in regions engaged when reflecting about one's own thoughts and beliefs (in the mPFC/vmPFC) when mentalising about those of socially close vs. distant others (Ochsner et al., 2004; Ames et al., 2008; Jenkins et al., 2008; Rabin and Rosenbaum, 2012; Rabin et al., 2013).

**Table 1:** **Selected studies showing increased neural activity in the self-other overlap when observing/inferring feelings of socially close vs. distant others**.

**Study**	**Feeling**	**Contrast**	**Self-other overlap**	**Paradigm**
Singer et al., 2006	Pain	Fair > unfair others	AI and ACC	Cues for shocks to others
Xu et al., 2009	Pain	Racial ingroup > outgroup	ACC	Pain to others (visual stimuli)
Cheng et al., 2010	Pain	Loved one > stranger	AI and ACC	Pain to others (visual stimuli)
Hein et al., 2010	Pain	Football team ingroup > outgroup	AI	Helping others in pain
Azevedo et al., 2013	Pain	Racial ingroup > outgroup	AI	Pain to others (visual stimuli)
Contreras-Huerta et al., 2013	Pain	Racial ingroup > outgroup	AI and ACC	Pain to others (visual stimuli)
Beeney et al., 2011	Social rejection	Close > distant friend	ACC	Ball toss exclusion game
Meyer et al., 2013	Social rejection	Friend > stranger	ACC and AI	Ball toss exclusion game
Mobbs et al., 2009	Reward	Self-similar > dissimilar other	VS and vmPFC	Card guessing game
Braams et al., 2014a	Reward	Self and friend > antagonist	VS	Gambling task
Braams et al., 2014b	Reward	Friend > stranger	VS	Gambling task
Molenberghs et al., 2014	Reward	Game ingroup > outgroup	VS and vmPFC	Giving money to others
Varnum et al., 2014	Reward	Self > friend	VS	Card guessing game

AI, anterior insula; ACC, anterior cingulate cortex; vmPFC, ventromedial prefrontal cortex; VS, ventral striatum.

Perceptions of future selves appear to share characteristics with perceptions of others. Pronin et al. (2008) found that people predicted their preferences in the future would resemble those of others more so than their own preferences, suggesting that future selves are perceived as socially distinct others, i.e., along a continuum of social distance. In a study by Ersner-Hershfield et al. (2009), photographs of participants were age-processed to make them look older. Showing participants photos of their future selves was intended to make the future selves more familiar to participants, thus making them socially closer. This task manipulation was found to cause preferences for delayed rewards to go up. Similarly, in a set of studies by Bartels and Rips (2010), participants were asked to rate how socially close they felt to future selves (operationalised by the authors as “psychological connectedness”.) Participants felt socially closer to future selves who were nearer in the future, and the closer they felt to future selves, the more they preferred delayed rewards in a temporal discounting task.

In a novel task by Jones and Rachlin (2009), participants performed a “social discounting” task in which participants chose between rewards for themselves or larger rewards for others across a range of social distances. The authors observed that people who preferred larger rewards for socially distant others more than smaller rewards for themselves were the same people who preferred delayed rewards in a temporal discounting task. This indicates that people's perceptions of the social and temporal distance of reward recipients are closely related, corroborating the findings of Bartels and Rips (2010).

To sum up, these findings collectively suggest that as the delay of a reward increases, the social distance of the future self recipient increases, and this corresponds with a decrease in the subjective value of rewards for them. As mentioned, studies of simulating others have shown that increasing the social distance of others reduces SE for them. If this effect applies to future selves, then increasing their social/temporal distance (by increasing reward delay) should reduce SE for them also. These delay-induced reductions in SE for future selves might lead to rewards for them being valued less, thus explaining why rewards are discounted with delay. This is the first critical mechanism of the simulation-based model of intertemporal preferences (SMIP), of which social distance is a key parameter.

The idea that simulation of others is analogous to simulation of future selves, and that the proximity of others/future selves modulates this simulation, has previously been put forward by Jamison and Wegener (2010). Here, we extend this idea, going into greater detail on the psychological mechanisms involved.

### Effects of social distance on simulation accuracy

Interestingly, compared to SE, social distance appears to have the opposite effect on SA (i.e., social distance appears to facilitate the suppression of egocentric bias). Recently, Tamir and Mitchell (2013) showed that when predicting the preferences of others (in terms of attitudes, hobbies etc.), egocentric bias in the form of self-similar responses was elicited only by socially close (i.e., similar) others. This finding suggests that in contrast to SE, increasing social distance increases SA. Savitsky et al. (2011) observed a similar effect using the Director task, finding that participants were less able to disengage from their own visual perspective when the director was socially close compared to distant. These two studies suggest that for socially close others, egocentric errors arising from simulation are more prevalent than for distant others. The ability to suppress egocentric biases is thus higher for more socially distant others, suggesting that SA is higher for more socially distant others.

The findings mentioned in the previous section demonstrate a clear relationship between SE and intertemporal choices, i.e., higher SE is associated with greater preference for delayed rewards. It is less clear how SA for future selves might affect these preferences, but a reasonable proposition can be formulated. In intertemporal choices, the self perspective (favored by egocentric bias) is that of the immediate self. From this perspective, delayed rewards have to be waited for; a cost that diminishes their subjective value. This cost-of-waiting does not exist from the perspective of future selves who are at the right point in time to receive delayed rewards instantly. Egocentric bias in intertemporal choices can therefore be considered to reduce preferences for delayed rewards. The ability to suppress this bias should see an increase in preferences for delayed rewards. As discussed, the ability to suppress this egocentric bias (consequently, SA) increases for more socially distant others. Extending this to future selves, increasing the social/temporal distance of future selves (by increasing reward delay) should increase SA for them, consequently increasing preferences for delayed rewards. This is the second critical mechanism of the SMIP.

Investigations of the neural correlates of ToM have identified a potentially important brain region for SA. Correct responding in false-belief ToM tasks relies on egocentric bias suppression skills, and these responses are reported to robustly increase activation in the right temporo-parietal junction (rTPJ) (Saxe and Kanwisher, 2003; Brass et al., 2009). Transcranial magnetic stimulation disruption of rTPJ processing causes ToM task performance decrements (Costa et al., 2008; Young et al., 2010), and enhancing rTPJ processing using anodal transcranial direct current stimulation improves Director task performance (Santiesteban et al., 2012). These studies suggest that neural processing in the rTPJ during false-belief ToM judgements is related to SA.

In keeping with the SMIP's assumption that SA increases with social distance, two studies have reported increased neural activity in the rTPJ for strangers compared to friends when inferring the pain of others (Cheng et al., 2010), and when making reward choices on their behalf (Braams et al., 2014b). Neural signals related to SA thus appear to be more readily elicited by socially distant compared to close others, in keeping with the predictions of the SMIP.

In spite of the central role of SA in intertemporal choices proposed by the SMIP, the rTPJ has not been commonly cited as a neural correlate of temporal discounting. One possible reason for this is that compared to false-belief ToM tasks, where SA is required throughout, temporal discounting trials require SA (and related rTPJ processing) in only a narrow subset of trials. In many temporal discounting trials, people can easily state preferences using readily available personal heuristics, e.g., “would you prefer £99 now or £100 in a year?”—this small difference in value could easily be recognized as not worth the wait. SA processes of the rTPJ might only be engaged when preferences are difficult to state and require additional information on predicted future states. fMRI studies have investigated this issue by comparing trials in which preferences were difficult vs. when they were easily stated, and in these contrasts increased activity in brain regions encompassing the rTPJ is reported, in the right intraparietal sulcus (Monterosso et al., 2007; Meade et al., 2011), inferior parietal cortex (Stoeckel et al., 2013), and angular gyrus (Hoffmann et al., 2008). On the basis of these findings, it is reasonable to infer that when decision-making requires information about future affective states, involvement of brain regions related to SA is observed. We note, however, that this line of argument based on fMRI data uses reverse inference, and is only mentioned here to address potentially similar reverse inferential criticism of the model (e.g., if the rTPJ is involved in simulation, and simulation is involved in intertemporal discounting, then why is the rTPJ not commonly reported as a neural correlate of intertemporal discounting).

In unpublished data (O'Connell et al., in-preparation), we used fMRI to measure individuals' magnitude of activity in the rTPJ during false-belief ToM judgements, which has been positively related to performance accuracy in this task (Gweon et al., 2012; Dodell-Feder et al., 2014). This putative index of SA was found to be higher in people with greater preferences for delayed rewards in a temporal discounting task, directly supporting the predictions of the SMIP.

### Simulation-based model of intertemporal preferences (SMIP)

From the discussed studies, two effects of social distance on simulation of others can be identified. Namely, increasing the social distance of others reduces SE for them (Effect 1), and increasing the social distance of others increases SA for them (Effect 2). We have also discussed evidence from studies of intertemporal choice suggesting that delay increases the social distance of future selves, allowing Effect 1 to be readily applied to intertemporal choices. Above, we postulate how Effect 2 operates in intertemporal choices. In contrast to Effect 1, Effect 2 leads to the counter-intuitive prediction that increasing the delay on larger-than-immediate rewards increases certain aspects of its subjective value, i.e., by reducing perceived costs-of-waiting via increased SA.

**Figure 2:**
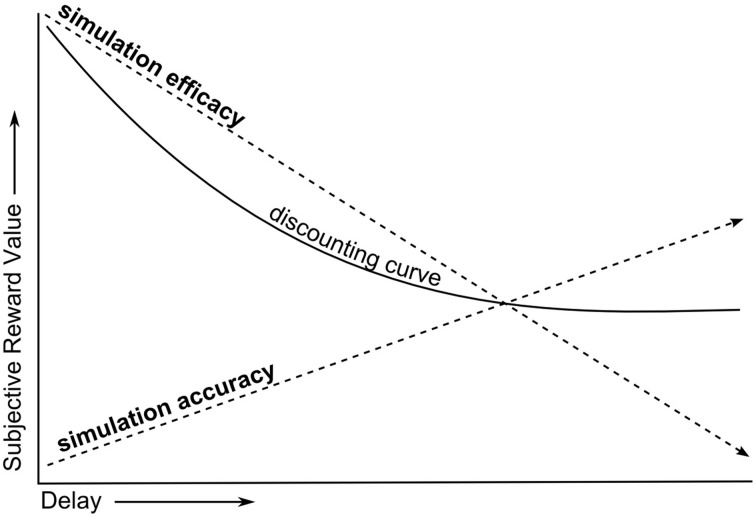
**Heuristic of SE and SA effects on temporal discounting**.

Put more simply, the SMIP explains the temporal discounting phenomenon as the result of two opposing forces of simulation on the subjective value of delayed rewards (Figure 2). In Effect 1, increasing the delay of rewards decreases SE for future selves, reducing the subjective value of delayed rewards. In Effect 2, increasing the delay of rewards increases the suppression of egocentric bias (and hence increases SA) for future selves, increasing the subjective value of delayed rewards. Since temporal discounting results in a net reduction in the subjective value of rewards with increasing delay, this model must assume that the rate at which Effect 1 reduces subjective value is greater than the rate at which Effect 2 increases it. Note that while SE and SA are oppositely modulated by social distance, they both share a positive relationship with preferences for delayed rewards.

The SMIP makes readily testable predictions. If validated, it would provide the delay-sensitive mechanism to empathizing with future selves in intertemporal choices proposed by Loewenstein (1996). The model's central idea that the ability to simulate governs intertemporal preferences has an appealing experimental upshot; temporal discounting might be used to index individual differences in simulation capacities. This prospect is appealing for the following reasons:

1. Existing measures of the ability to simulate others are often only sensitive to one component, either SE (e.g., self-other pain paradigm), or SA (e.g., false-belief ToM tasks, the Director task). The SMIP suggests that individual temporal discounting functions are the product of both components, thus providing a composite empirical metric of simulation capacity.
2. The subjective value of amounts of money can be parameterised by their objective worth. This feature of monetary versions of temporal discounting tasks provides a means for standardizing subjective experiences, which allows for tight comparisons between individuals in their ability to simulate others by mapping the simulated feeling (of value) on a monetary scale.
3. Practically, the temporal discounting task is fast (~8 min), easy to perform (simple reward choices), and makes low demands on verbal abilities, all of which allow it to be used effectively across a wide range of developmental and clinical populations.

One prediction of the hypothesis that rewards are temporally discounted because of reductions in SE for future selves (Effect 1), is that people should discount rewards more rapidly when SE is low, as is supposedly the case when simulating socially distant others. As briefly mentioned, we provided support for this hypothesis by showing that people discount for distant others more steeply than self or close others (O'Connell et al., 2013). Individuals who scored high in trait empathy were also found to discount less for distant others compared to those who scored low. Note that trait empathy has been positively associated with SE in terms of neural responses in the self-other pain paradigm (Singer et al., 2004; Akitsuki and Decety, 2009). These findings indicate that trait empathy is a correlate of SE, suggesting the possibility that in our results, temporal discounting for distant others was affected by individual differences in SE.

Two further lines of research deserve mention in their contribution to the evidence-base for the SMIP, and their potential importance for future evaluations of the model.

### Development of ToM and intertemporal preferences

Initial work directly testing the relationship between ToM and intertemporal preferences came from Thompson et al. (1997). Their results showed that in four-year-old children, accuracy in the false-belief ToM task was positively related to preferences for delayed rewards (stickers). In a similar recent study by Marchetti et al. (2014), temporal discounting (with sweets) was found to be more predictive of performance on the ToM task than age, with children who were more patient scoring better on a first order false-belief task. These findings are in line with the SMIP's assumption that a greater SA capacity (which involves a greater capacity to inhibit egocentric bias) for others extends to future selves, promoting the subjective value of rewards for them. However, Metcalf and Atance (2011) found only a marginal relationship between ToM task performance and the ability to delay gratification in children aged between 3 and 5 years old. In summary, developmental studies largely show a positive relationship between false-belief ToM accuracy and preferences for delayed rewards.

### ToM and intertemporal preferences in neuropsychiatric conditions

People diagnosed with Autism Spectrum Conditions (ASC) are marked by deficits in false-belief ToM task performance (Baron-Cohen et al., 1985), possibly due to a reduced ability to simulate others (Oberman and Ramachandran, 2007). The current model predicts that such simulation deficits should cause people with ASC to discount delayed rewards more steeply than neurotypical controls. Although reward processing has been widely studied in ASC (Chevallier et al., 2012), surprisingly there is no available temporal discounting data on adults diagnosed with ASC. Three studies have measured temporal discounting in children and adolescents with ASC, one finding evidence of more impulsive choice preferences in ASC (Chantiluke et al., 2014), and two non-independent studies reporting no evidence of abnormal discounting compared to typically developing controls (Demurie et al., 2011, 2013). However, it should be noted that in these latter null findings, the longest delay used (2 weeks) was much shorter than is common in temporal discounting tasks (>6 months). Such short delays might not tax temporal discounting-related processes enough to flag-up potential abnormalities in ASC.

Another clinical condition marked by deficits in ToM is schizophrenia (Brüne, 2005; Bora et al., 2009). Shamay-Tsoory et al. (2007) have reported that ToM deficits are more pronounced for inferring the feelings vs. beliefs states of others in this group. In addition, the volume of the vmPFC—a brain region implicated in simulating the mental states of others (Shamay-Tsoory et al., 2006; Shamay-Tsoory and Aharon-Peretz, 2007)—has been positively related to scores on a range of ToM measures in people with schizophrenia (Hooker et al., 2011). These findings suggest that the ability to infer the feelings of others is disrupted in people with schizophrenia. According to the SMIP, such a deficit would be expected to coincide with steeper temporal discounting, which has been consistently reported in people with schizophrenia (Heerey et al., 2007, 2011; Gold et al., 2008; Ahn et al., 2011).

## Summary

In this article, we examine the role of simulation, as used to infer the feeling of others as well as our future selves, in making intertemporal choices. Specifically, we propose how two distinct components within simulation, simulation accuracy (SA) and simulation efficacy (SE) influence intertemporal choices. These components are influenced differentially by social distance, but are similarly modulated by delay when making choices for future selves. The resulting theoretical framework, called the SMIP, lays out clear empirical predictions. If these predictions are validated, the SMIP can lead to new lab-based measures to characterize the social cognitive deficits in psychopathological conditions such as ASC and schizophrenia.

### Conflict of interest statement

The authors declare that the research was conducted in the absence of any commercial or financial relationships that could be construed as a potential conflict of interest.
